# Exploring Older Adult’s Views of the Age-Inclusivity of Physical Activity Websites Using the Think Aloud Method: Qualitative Analysis

**DOI:** 10.2196/68951

**Published:** 2025-06-19

**Authors:** Veda Clemson, Elisabeth Grey, Julie Barnett, Ella Burfitt, Fiona Gillison

**Affiliations:** 1 Department for Health, University of Bath, Claverton Down, Bath, BA2 7AY, United Kingdom, 44 1225 384387; 2 The National Institute for Health and Care Research Applied Research Collaboration West (NIHR ARC West), University Hospitals Bristol and Weston NHS Foundation Trust, Bristol, United Kingdom; 3 Population Health Sciences, Bristol Medical School, University of Bristol, Bristol, United Kingdom; 4 Department of Psychology, University of Bath, Bath, United Kingdom

**Keywords:** ageism, inclusivity, digital literacy, older adult, physical activity

## Abstract

**Background:**

Older adults are the least active in our society and may face additional barriers to taking part in physical activity compared with those experienced by younger people because of factors such as lower digital literacy and negative stereotypes of aging.

**Objective:**

This study aimed to explore how older adults navigate websites that provide access to physical activity opportunities and facilities and make judgments about their suitability.

**Methods:**

Semistructured interviews were embedded within a think-aloud approach. Participants were shown a series of websites and asked to navigate through the websites as if they were going to take up what was on offer, articulating their thoughts and comments out loud as they progressed. Participants viewed up to 4 websites, rotated from a pool of 8, including leisure centers, exercise products, gyms, or community organizations. Additional questions were asked about perceptions of the inclusivity of the websites at the end of the interview. Digital recordings were made and transcribed verbatim, and analyzed using thematic analysis.

**Results:**

Nineteen participants (6 male and 13 female) aged between 65 and 84 years were recruited from southern England; one-third reported having poor digital ability prior to taking part. Three overarching themes relating to the research question were identified as follows: (1) signals of age-inclusivity, (2) limiting beliefs, and (3) confidence in making judgments. Older adults inferred a lot of information about how welcome they would be in physical activity settings from the images and language used on websites. They showed a preference for imagery that was inclusive of age, body shape, and physical ability, not only for those depicting older adults themselves. Some adults reported firm views about the type and intensity of physical activity that is appropriate for older adults, and many expressed a specific dislike of gyms, based on both the (young) age of most users and perceived emphasis towards aesthetic rather than health-related exercise. While most participants could navigate websites successfully, they preferred to visit venues and speak to staff to gain greater confidence that they would feel welcome and that the activities would be at a suitable level.

**Conclusions:**

Websites providing access to physical activity could be more inclusive of older adults by using more diverse imagery, providing clearer descriptions of the activities on offer, along with details of the level of fitness or ability needed to take part, and providing alternatives to web-based booking. Additional societal-level approaches to reducing age-limiting self-stereotyping may also be useful in expanding the opportunities for older adults to access mainstream provisions for physical activity.

## Introduction

Older adults are the least active in our society [1], yet they could derive significant benefits from physical activity in terms of preventing and managing chronic disease, promoting strength and function [2], and reducing morbidity [3]. The UK government recommends 150 minutes of moderate-intensity activity per week for older adults, as well as breaking up sedentary periods and including exercise that improves muscle strength, balance, and flexibility [4,5]. Data for 2022 to 2023 from Sport England estimate that around 53% of people older than 65 years achieve this, with 36% achieving less than 30 minutes of activity per week [6]. Activity in later life reduces the risk of disability, the development of noncommunicable diseases, and the decline in functional capacity associated with aging [7]. There may also be benefits to cognitive health [8].

Older adults experience many of the same barriers reported by younger adults to physical activity, including time, cost, and enjoyment, and lack of confidence or fitness [9,10]. Additional barriers that are more commonly seen in older adulthood relate to physical function, such as ill health or disability, safety concerns, and fear of falling [10]. However, there are also indirect barriers that may influence engagement with facilities and activities in older age, including poorer digital literacy, digital exclusion [11], and negative stereotypes around aging [12]. Considering digital literacy, while many older adults do own smartphones or other digital devices, digital acceptability and access decline with age [13]. The physical activity industry mirrors the rest of society in having become more digitized, for example, through moving to web-based bookings, payments, and advertising, and the development of increasingly technical exercise equipment. These changes have an unintended negative impact on access for older adults.

Older adults face several age stereotypes relevant to the physical activity domain, often being portrayed as frail, easily fatigued, and physically inactive [14]. Some of this stereotyping can be observed in how older adults are treated by others, for example, fitness professionals commonly have (and communicate to greater or lesser degrees) low expectations of older adults’ capabilities, which in turn discourages older adults from taking part [15]. Providing positive examples of the capabilities of older adults that present a very high level of ability (ie, exceptional fitness) can also be problematic to some older adults who feel they may not be able to match up to expectations, putting them off taking part [16]. Age stereotypes for physical activity are not only held by younger adults, older adults themselves often share these negative perceptions, for example, feeling that their aging body means they are less able to exercise, and that people their age are out of place in physical activity settings, such as gyms and health clubs [17].

The theory of stereotype embodiment sets out one way in which self-stereotyping may develop, proposing that people internalize negative stereotypes throughout their lives, developing negative attitudes toward older adults, which are directed toward themselves as they age [18]. This leads to unconscious effects on behavior, particularly in settings where age stereotypes are primed (eg, depictions of older adults with limited ability), impacting health behaviors [19]. Little research from this perspective has been undertaken specifically in relation to physical activity, and research exploring the impact of age stereotyping on physical activity from other theoretical perspectives has resulted in some conflicting results. For example, in response to age-based stereotype threats (ie, pressure to avoid confirming or exacerbating stereotypes when taking part in an activity; [20]), some studies show poorer performance in physical activities, such as walking speed [21], while others show improved performance, for example in fitness training [22]. Thus, research to provide greater insight into older adults’ responses to how they are portrayed during their first approach to physical activity settings is warranted and could help us to understand the variation in effects.

The aim of this study was to explore how websites providing information about physical activity opportunities may influence the barriers or facilitators of physical activity for older adults. Using a “think aloud” approach, our objectives were to (1) explore the social signals older adults identified of inclusivity, fit, or welcome, and (2) capture their views about the digital capability required to find relevant information. Together, this would provide information on how older adults experience web-based gateways to access physical activity opportunities and what information they draw on in assessing whether the activity on offer was suitable for them.

## Methods

### Ethical Considerations

Ethics approval was granted by the Research Ethics Committee for Health at the University of Bath (EP 20/21 105). All participants provided informed written consent, and any identifying information was anonymized prior to data analysis. Interviews were conducted between January 2022 and August 2023.

### Design

This study was situated within a constructivist research paradigm, selecting a “think aloud” approach in order to explore and understand people’s subjective experiences and focus on meaning-making in real time [23]. The think aloud approach has shown previous utility in exploring human-computer interactions and decision-making [24] and the usability of websites in health settings [25].

The approach encourages participants to verbalize their thoughts or feelings while navigating web-based resources. In our study, this was embedded within semistructured interviews to ensure interviewees had been asked comparable questions when interpreting the findings, and to allow participants to express their thoughts and experiences at a more general level [26]. The interviews were all conducted in person by slim, White, young women (aged 18‐23 years); we include this detail as it was commented on by some participants, so it may have influenced some of their responses.

### Participants

For this study, we considered adults to be aged 65 years or older, in line with the UK Office for National Statistics [27]. The inclusion criteria were individuals aged 65 years and older, who can read and speak fluent English, self-assessed as physically able to, and interested in taking part in physical activity. Participants were recruited through advertisements posted in local community centers, on local social media platforms (such as “Nextdoor”), and through the authors’ existing networks. We aimed to recruit 20 older adults to provide a range of experiences, focusing on advertising in lower socioeconomic areas. Participants received travel expenses and refreshments but no payment for their time.

### Procedure

Volunteers were sent a participant information sheet and consent form and invited to set up a time for their interview; these arrangements were typically made via email, but could be by phone or letter if preferred. Interviews were conducted in public spaces of the participants’ choice (to ensure they would feel comfortable) using a laptop provided by the researcher. Participants were asked to nominate a café local to them or could choose to come to the University. At the start of the interview, the researcher talked through the process and obtained written consent. The researcher then presented between 1 and 4 example websites in turn, working through the think aloud process with each until the participant wished to stop or the 1 hour had passed; participants were offered the choice whether to continue at the end of each website viewing. The order in which websites were presented was rotated to ensure that the findings related to a variety of website designs and topics.

Participants were encouraged to provide an honest opinion on the websites, add any thoughts which came to mind [28], and were prompted to reflect on what they were doing as they browsed (eg, “why did you click on that?”; Multimedia Appendix 1 contains the full topic guide). In line with a relaxed think aloud approach, the interviewer only interacted with the participant when asking prompts or in response to a participant’s request for help with navigating the websites [28]. At the end of browsing websites, participants were asked to evaluate the content overall. One example of the questions asked for this was “were there any aspects of the websites that made you feel included or excluded?”.

The pool of example websites was chosen to reflect different aspects of the physical activity industry following public engagement activity with 7 older adults as part of a related study (ie, what they consider as part of the industry), and discussion with an advisor from a national representative body. Our aims were to include both commercial and noncommercial services, sites that advertise equipment as well as facilities, and ensure we featured a range of activities that are accessible to someone starting out (eg, activities that do not require extensive technical skills, and can be started at low intensity). The research team reviewed the final set to ensure we included variations in website designs (Multimedia Appendix 1 lists the specific websites chosen). Two examples were available from each of the following 5 categories: local authority providers of physical activity services; fitness industry providers (eg, generalist gyms that focused on building strength and endurance); fitness industry product providers (eg, personal fitness equipment and clothing); community organizations that encourage physical activity (eg, walking groups); and UK physical activity campaigns. The order in which the websites were presented was rotated.

After the interview, participants provided information on their demographics, usual physical activity level, and self-rated health.

### Data Analysis

Interviews were audio-recorded using a digital voice recorder, transcribed verbatim by VC, and anonymized. Analysis was conducted using a 6-step thematic analysis [29], including familiarization, generating initial codes (including a code book), organizing codes into clusters of shared meaning (themes and subthemes), and refining and naming the themes with the wider research team. Two transcripts were coded by VC and FG to refine the codebook, and the remainder were then coded by VC and the themes were developed iteratively.

Website tracking software was used to generate a recording of participants’ progress in order to verify which specific pages participants were looking at as they spoke. This information has been added in square brackets within quotes where appropriate.

## Results

### Sample

Participants (6 male and 13 female) were aged between 65 and 84 years and based in Southern England. Fourteen (74%) participants had postschool qualifications, and the majority of the participants considered themselves to be healthy (95% scored >7 on a 10-point scale), with moderate fitness (most reported a steady or brisk walking pace) (Table 1).

Around a third of participants considered that they had poor digital ability, but the majority were able to navigate the websites without difficulty, and the challenges experienced most came from using unfamiliar hardware (ie, using the researcher’s laptop, rather than a usual PC at home) rather than difficulty using common website functions (scrolling down, search function, clicking through different pages). At the extremes, 2 participants were not confident in navigating websites unaided, so they asked the researcher to do this for them (P11 and P18), while one participant was a website designer prior to retiring (P4).

Three overarching themes relating to the research question were identified as follows: (1) signals of age-inclusivity, reflecting what participants read into the images and language used on websites; (2) limiting beliefs, reflecting how participants related beliefs about their own physical capability, and age stereotypes to website content; and (3) trust, reflecting older adults’ reservations about what could be understood from web-based information alone.

**Table 1.** Participant characteristics.

	Age group (years)	Gender	Ethnic group	Highest educational attainment	Usual walking pace	Health rating^a^
1	65‐69	Man	White	Bachelor degree	Steady	8
2	80‐84	Woman	White	Bachelor degree	Brisk	7
3	75‐79	Woman	White	Master or postgraduate qualification	Brisk	7
4	65‐69	Woman	Black or African	GCSE/O level^b^	Slow	3
5	75‐79	Woman	White	Master or postgraduate qualification	Steady	8
6	70‐74	Man	White	GCSE/O level	Brisk	8
7	65‐69	Man	White	Bachelor degree	Steady	7
8	70‐74	Man	White	Master or postgraduate qualification	Steady	8
9	75‐79	Man	White	Master or postgraduate qualification	Steady	7
10	65‐69	Woman	White	Master or postgraduate qualification	Steady	7
11	65‐69	Woman	White	Bachelor degree	Brisk	8
12	65‐69	Woman	White	Bachelor degree	Steady	9
13	75‐79	Woman	White	Master or postgraduate qualification	Slow	10
14	65‐69	Woman	White	Master or postgraduate qualification	Steady	9
15	65‐69	Woman	White	No formal qualifications	Slow	8
16	65‐69	Woman	White	Master or postgraduate qualification	Brisk	8
17	80‐84	Woman	White	A Level or equivalent	Steady	7
18	75‐79	Woman	Asian or Asian British	No formal qualifications	Brisk	7
19	65‐69	Man	Black or African	Master or postgraduate qualification	Slow	8

a Self-rated on a scale of 1‐10 where 1 is very bad health and 10 is excellent health.

b GCSE/O level: General Certificate of Secondary Education Ordinary Level.

### Theme 1: Signals of Age-Inclusivity

#### Images

Participants drew on images, language, and the type of physical activity or service offered in judging whether websites were relevant to them. Images were often the first source of information that participants considered (“I noticed almost immediately there was a mix you know, that in photos there were older people*”* [P9]) and were often taken very literally as an indication of who uses a particular facility. Participants extrapolated from who was depicted as to whether they would be welcome as a participant:

*This is the gym… oh… group exercise... oh let’s see if there’s any for old people. [inspects the photograph]. There’s a young lady… that’s not for me! Absolutely not for me. If they had a photograph of a group with a couple of older people involved in the same group yep, I don’t mind being with young people at all, except I wouldn’t like to be the only one.*

[P2]

*It’s modelled by people, young people without wobbly bits.*

[P15]

While seeing older adults depicted was interpreted as a clear indicator that a website was offering something intended for them, most participants used a broader benchmark and judged websites as age-inclusive when they saw images of people of different ages, genders, races, and abilities, as well as people with disabilities. For example, on articulating why certain websites looked age-inclusive, participants stated:

*[Because] they’re all normal people, as in, of an age that I can relate to. So it’s not, it’s not big punchy “do this to build your muscle” type thing it’s all about… it’s all about keeping active and keeping healthy through, through activity, which is good.*

[P6]

*That one is, I think that’s very, the seems quite welcoming that, I’d have a look at it, I would if I saw that picture... Because it’s a family there, and there’s a child there, it is not as though someone’s just exercising and looks really fit (laughs). I mean, I’m not saying they don’t look fit but I mean it’s not a real over-fit.*

[P17]

In addition to images, participants responded positively to clear statements that all ages are welcome:

*Um… they [community rowing club] particularly stress the headline [ages] 13 to 100 so, it definitely was not focused just on the… you know the normally you associate the, I suppose I associate rowing with the boat race, young fit men or women from um from the Universities etc. That’s my first reaction so um this seemed quite inclusive in that sense, quite friendly, seems quite informative.*

[P9]

Similarly, images of people of all body sizes were considered positive and inclusive, regardless of their age. As such, body size appeared to serve as a proxy for participants believing an activity would be achievable for them:

*But I mean, but you see that’s still good to get people going isn’t it because they’re all shapes and sizes [in the image] and I’d rather do classes with people like that then just really old people so that’s quite nice*

[P17]

*They are… women and they are older women, kind of thing that they are not… slim ones so they really appeal to the likes of me that it can be me, joining them, you see.*

[P7]

While wanting to see older adults depicted, participants could be very particular in how old, or how active the older adults were. Some interviewees distanced themselves from websites where older adults were depicted as being too old or inactive:

*They are very old people and they don’t appeal to me at all. I couldn’t interact with anybody like that. I mean, okay, they might get satisfaction out of it but nah. It’s not me, it’s just not me.*

[P15]

Very few older adults expressed an interest in exercise sessions that are solely available to older adults, preferring to see indicators of inclusivity to the whole community, with older and larger people depicted as part of this. This may reflect (as suggested by the quote from P15) the degree to which participants have actively embraced an older identity.

#### Language

In some cases, the language used on websites signaled to participants that these services or facilities were not for them. This was particularly evident in websites relating to gyms and exercise classes, which included new “branded” terms such as classes called “SHRED,” “Trax,” and “Rush”. Participants viewed this terminology as an immediate turn-off, and an indicator that such activities were not for them:

*It’s delving into a sort of more modern language that I don’t necessarily understand, but that’s probably more my problem than anybody else’s!*

[P6]

*Oh… what are glute exercises? Oh… glute, did you know about glute muscles? I didn’t*.

[P13]

*Shred (laughing)… see I, well I’m just looking I’m thinking right, so I immediately focused in on classes and I’m not going to go and do a class that says shred because um I just know that that is just, you know, I wouldn’t do it.*

[P12]

Some participants were explicit in describing the lack of explanation of such classes as discriminatory:

*I don’t think for my particular age group that they are informative enough and I find that they are a bit discriminative and they’re… ageist. That’s it.*

[P15]

### Theme 2: Limiting Beliefs

#### Perceived Physical Capability

Most participants recognized and described a deterioration in their physical ability as they had aged and expressed a desire to avoid injuries by attempting to do too much. While some participants had long-term conditions such as arthritis that limited their activity, for many, this reflected a fear of injury based on the perceived limitations of age alone. This caution influenced their responses to websites based on the type of exercise on offer:

*With the equipment, I’d end up… tearing something or ending up injuring something of my body. So, I’ve come to the conclusion that you know, probably it’s best not to be going into gyms.*

[P4]

This concern was also reflected in participants’ desire for more information about the intensity of activities than was provided on most websites. This is often related to expected levels of fitness and capability to start engaging in the exercise on offer, reflecting the fact that although they might have been experienced in some activities (ie, not a beginner), they knew they were much less fit than when they last took part. For example, 2 participants commented on the website of a local walking group that was marketed towards older adults, and depicted older adults in their imagery:

*It would be helpful if there was a bit more information, to give you… the severity of the walk, is it rough terrain or not. Because obviously as you get older, the flatter the walk the more… probability that you’ll actually go onto it…*

[P6]

*Older people do tend to walk a little bit slower so you want to know is this going to take me a couple of hours, or is this going to take all day?*

[P6]

*My concern would be walking out for the first time … I’d be worried about lagging behind and upsetting people who are experienced walkers. If they had a ‘break me in walk’ and then oh you are broken in a bit now so you can do the next one, and the next one.*

[P15]

Similar concerns were offered about fitness classes:

*It [the website] didn’t exclude me, it’s just I, my body and everything else that comes with it, makes me feel… a bit worried to try to go and lift weights or do some strenuous thing that I know my body can’t take… Do I need to be a certain fitness level before? That’s something I need to know.*

[P4]

Others expressed concerns about potentially embarrassing themselves in mixed settings or pushing themselves too far. For example, on looking at a strength training class video, one participant commented:

*If you’re not fit I don’t think you would ever look [to go] there because you would feel such a fool. You would, you would think I’m a wimp, I couldn’t cope with that, so I wouldn’t go.*

[P17]

Some older adults also expressed concern that younger instructors or trainers would not be able to adapt to what was needed for older adults, and thus that what was demanded of them would be too great. These participants tended to use the age of instructors depicted on websites as a signal of whether a service or facility was appropriate for them:

*I don’t know whether I’ve just got more respect in general for older people because they’ve got more experience, maybe that, I just don’t quite believe that someone – sorry about this* [referring to the young age of the interviewer] *– does a three-year course in whatever, fitness, can necessarily have all the information they need to help older people.*

[P11]

*[I’d need] way more support, but who’s going to be supporting me, I mean, some squeaky chicky ain’t going to come over and say ‘*Alright, old love let’s sort you out!’

[P15]

Preference for older instructors was not a universally held view, and those who had more experience exercising in mixed-age groups provided examples of younger instructors who adapted classes well. They also flagged the need to differentiate people by ability rather than age:

*My Pilates class is basically for over 50’s but they want to stop it too because they say people don’t like being defined by their age… so it’s like how do you find another way of saying over 55 without patronising somebody?... But at the same time sometimes you don’t know what you’re looking for. There’s some people who are over 65 and they don’t need to look at an over 65 sort of over 50s class because they’re really fit and they can still do stuff in an ordinary class*.

[P12]

#### Stereotyping

While participants were generally pragmatic and objective in acknowledging age-related changes in physical capability, there was also some evidence of stereotypical thinking. Some of this appeared to relate to self-stereotyping of what types of physical activity older adults should be doing. The example below relates to a comment made while reading through a list of exercise classes at a municipal leisure center.

*... I’d go for body balance but yoga, no, spin, sounds too fast, those aren’t right for old people… If its high intensity, we [older adults] shouldn’t be doing things high intensity.*

[P2]

However, many of the interview excerpts coded into this theme related to gyms. It was often hard to disentangle participants’ pre-existing attitudes towards gyms as a type of business (and their experience of them being predominantly used by young people), from the specific aspects of the website presented (“I’m actually a little bit against gyms anyway, because, it’s a modern thing*”* [P7]). These views typically reflected participants’ attitudes towards gyms rather than what was presented on a website, as well as their experience of gyms.

*I didn’t like going in the actual gym bit because it was all people a lot younger than me and they were all trying to outdo each other.*

[P15]

*I see these [gyms] and I only tend to see young people. You know, cool looking sort of young people going into (particular gym), and that doesn’t, that doesn’t do anything - this website - to disabuse me of that viewpoint. It comes across as a younger, commercial first [place].*

[P9]

*I mean I wouldn’t go anywhere near that place [gym]. You know I, not being rude, but I mean cause to me that is just full of… people who are like nearly old enough to be my grandchild so I’m like just wouldn’t (laughing), so I wouldn’t go in there no.*

[P12]

In some cases, the stereotypical thinking reflected assumptions that gyms are a place people go to do image—rather than health—enhancing activity. Often, the negative statements about young people are conflated with assumptions about why people use gyms, given the greater prevalence of young people using them.

*It’s all a bit showy-off-y to me [going to the gym], rather than, I suspect… intended just to get you fit and keep you fit. It’s a bit, it’s all a bit show-y.*

[P7]

*When I have been into the odd hotel gym there’s generally some very, very narcissistic p- you know people there looking at themselves more in the mirror than they are [exercising], you know what I mean, it er, to a younger person you know a, beautiful blonde there or really fit young bloke you know, you know doing whatever I’m thinking, yep, you don’t need that*.

[P9]

Some participants were less critical of the reasons why people attend gyms, but in believing gyms are for building muscle, and that building muscle is something that only younger people do, they assumed that gyms were not for them. This was reinforced by images of young fit adults lifting weights:

W*hen you get over 65, you’re more interested probably in er… you’re not going to build muscle, you’re not going to, you know, attain any major goals. You want to, keep mobile keep flexible, and if there were more things to build upon flexibility, um… that would be more appealing to people of an older age group, yes*… *There doesn’t seem to be any of that on this website*.

[P6]

*They [images] all look quite frightening to me, because they all look so much younger and fitter! … I mean look at the, these guys are all pretty stacked, so. I’ll never look like that. Not that I ever did!*

[P6]

### Theme 3: Confidence in Making Judgments

Participants preferred websites where information was presented clearly on the first page, where the font was simple, without too many distractions from moving images. The majority considered information about the proximity of facilities (“See, straight away I think what I want to do is find out, where’s the nearest one for me?*”* [P6]), costs, and links to a person they could contact to be of primary importance. They wanted to be able to see this information without needing to click through multiple screens.

*I want to know what the membership is and it’s giving me “hear from our members.” I want to know where it is, how much it is, swimming pay as you go*

[P15]

*So this is, they’re not going to give me any information about courses until… until I open the portal and I’m not interested in that.*

[P5]

Even among those comfortable with using websites and digital tools, many participants expressed a preference to establish some personal contact before committing; they looked for a phone number of someone to talk to, or stated that they would not book via web, but would use the site to gather information and then visit the venue to book. For some, the need to verify information in person reflected a lack of trust in what is presented via web, rather than poor digital literacy.

It’s all a marketing ploy, not really giving me the information that I really want.

[P15]

*I… can’t tell, what they would be like from the website… but it’s all part of an advertise-advertising their product um so, so yo-you… that’s how I would expect it to be I’d expect it to be advertising a product I wouldn’t expect to see any truth there*

[P7]

This hesitancy may also reflect participants’ interest in factors that cannot be ascertained from a website, like the atmosphere of the venue or how they are treated by staff.

*If I was going to join the gym, I’d go down there and physically look at it. So, um, but only based on having… drawn up a-a-a-a list of 3 say [from their websites]… and that’s what I would do and then go and look at them, and see what I thought and judge it on, on um… the people that are um… are um running it so forth, do you get a welcome er a good welcome there, do they seem to know what they’re talking about*.

[P7]

Hesitancy about using websites to make decisions on the appropriateness of physical activity opportunities appeared to differ from general comfort with digital devices or web-based shopping. For example, P7, who would not trust the “truth” of a leisure center website, was comfortable with buying sports equipment via web (“I buy my sports stuff online, or off mates”). Others reported being able to use a device, but not being comfortable with website bookings as a specific function:

*Well I’m not very good at websites to be honest, and actually although I’m on it on my iPad with the gym, my son has to book me online all the time. I can’t do it, it’s sad init*?

[P18]

## Discussion

### Principal Findings

This study explored older adults’ reactions and views of the age-inclusivity of websites providing access to physical activity opportunities. Together, the themes showed how older adults drew on the imagery and language of websites to inform their decisions, as well as some existing schemas of what types and intensities of activity are appropriate for older adults (ie, implicit ageism). Participants were encouraged by websites depicting people of mixed ages, body shapes, and abilities using their service, and were not looking for services offered purely for older adults. Many older adults judged whether an opportunity was appropriate for them from factors that signaled the intensity and challenge of the physical activity on offer, motivated by a wish to avoid injury or embarrassment, and felt the ability to keep up with others. Information on the level of ability of fitness required to take part was rarely presented directly, which may have caused participants to rely on images and language to make these inferences. Participants showed a greater preference for and trust in websites that provided clear and up-front information on basic considerations such as cost, location, and contact details. Nonetheless, they reported still being reluctant to believe an opportunity was for them without having visited a venue and spoken to staff.

The think aloud approach has been used in previous research to explore how people use and make sense of websites, and what they base their judgments on [28]. For example, users have been shown to use short case examples to understand the relevance of the site by drawing constant comparisons between the examples presented, their own experience [28]. While in this study few websites that we used included case studies (although these were popular where they did), we did observe a similar effect in how participants were extrapolating information from the individuals shown in the images based on their experience and existing attitudes; for example, some participants inferred knowledge about a “typical user’s” motivation and fitness level just from a photo. This process was most closely observed in older adults’ responses to images of young people, using this information to form judgments that a facility or service was not for them. In part, this reflected participants’ explicit narratives of how their capability and motives for physical activity had changed over time, such that they judged what is suitable for young adults would not be suitable for them. While some settings (eg, gyms [30] and surfing [15]) are already considered a young adults’ domain by default, our study showed that in other settings too, seeing images predominantly of young adults was used as a heuristic for assessing an activity as irrelevant or inappropriate. This response was diminished when images included mixed age groups, ethnicities, and body shapes alongside young adults.

Past work has described how older adults who take part in physical activity need to navigate the tension between experiencing the benefits of physical activity, noticing their own physical decline, and dealing with social norms about what type of physical activity is acceptable in older age [30]. This tension appeared to be visible in our study, acting as a lens through which participants were evaluating whether the activities that the website was promoting were relevant and welcoming to them. In line with other work [10], we found that older adults’ beliefs about their capabilities were a common barrier to physical activity, and evidence of an apparent acceptance of the inevitability of physical decline [17]. Current health messaging, as well as marketing of physical activity facilities and opportunities, may assume that older adults are interested because they believe that there is scope for improvement, so exposing this alternative perspective—that older adults largely do not feel that improvement in strength and fitness is feasible—could be important to inform future approaches.

While participants in this study showed a good ability to navigate websites and good digital literacy, they still exhibited some lack of confidence in applying this. Two participants either asked for help from the Researcher in navigating the websites or reported that they normally ask a younger family member to make web-based bookings or purchases for them. This aligns with previous research demonstrating that even among those with high levels of competency in using websites, older adults may have lower perceptions of their usefulness [31] and acceptability [11]. In this study, this lower perceived usefulness may relate to the fact that information older adults consider crucial was not provided; websites rarely provided explicit statements about welcoming all ages, contact details, clarity about the level of starting fitness required, or information on how demands can be tailored. However, alternatives to web-based booking, whether by phone or in person, will likely remain an important part of providing an age-inclusive offer, to allow older adults to test out the atmosphere of a venue and sample interactions with staff.

### Strengths and Limitations

The use of think aloud interviews worked well in this setting by encouraging participants to verbalize their thoughts and experiences while interacting with websites in “real time.” It is argued that this often results in more honest accounts of user experience [32]. A limitation of this approach was that participants were navigating websites that they had not chosen for themselves, on a device that was not familiar to them. Participants may have been less distracted by the mechanics of navigating a website if using a familiar device. Our findings may also have been influenced by the specific websites we chose. While we attempted to mitigate this by identifying a range of different websites and rotating these through interviews, providing choice to participants (eg, allowing them to nominate the types of physical activity there were interested in) may have helped to provide a greater test of participants’ views on the inclusivity of the website, rather than the type of activity it offered. In particular, it was hard to disentangle participants’ comments on the desirability of gyms and the imagery that accompanied them, reported in theme 2, from their preexisting attitudes.

We attempted to recruit a diverse sample by advertising in areas populated by people of lower income and with greater ethnic diversity, and by seeking support from gatekeepers (eg, local Black and minority ethnic support groups for older adults). However, the majority of those willing to take part were White, highly educated, and living in urban areas, which is a limitation of this study. Other studies conducted with older adults report greater challenges with website navigation [25], which we may have found if we had recruited a sample with a more diverse socioeconomic background. Our participants were also relatively healthy; this is less a limitation and more a reflection of our design, as in order to address our research question, we recruited individuals for whom the websites were relevant, that is, those who are capable of engaging in physical activity. However, we may have discovered different barriers had we included more people with limited physical ability. Related to this, the age of our participants spanned around 2 decades from 65 to 84 years, from those still working to those who have been retired for 20 years. People at either end of this age group, as well as those experiencing greater or lesser physical decline or limitations, may have had different perspectives on who the websites depicted and what was offered. Within this small qualitative study, we were not able to tease out these differences.

### Implications and Future Directions

The findings of this study suggest that older adults are more likely to consider that services and facilities are relevant and attractive to them when websites depict a diverse set of users, including an explicit statement indicating people of all ages are welcome, and present clear information to users to judge the level of fitness and ability likely to be required to take part. Remaining physically active for longer is key to reducing age-related disability and disease [7] and promoting good cognitive health [8]. In addition, including older adults in intergenerational settings can foster the mental and physical health benefits associated with social inclusion and connection [33,34]. If increased physical activity participation in the growing population of older adults is to be achieved, their needs need to be accommodated affordably within mainstream facilities and services.

A common reason why older adults wanted more information on the likely demands of the physical activity on offer was to reassure them that they would not be pushed too far, feel embarrassed, and that the activity could be adapted safely for someone of their age and level of ability. Classifications referring to the level of experience (beginner, intermediate, etc) may not be meaningful when someone has historical experience but has not taken part in many years, and a lack of explanation alongside activities that have nontraditional names can lead older adults to infer that this is not intended for them. Evidence of venues using older adults as instructors and coaches, and information to confirm that activities can be adapted to be appropriate for all ability levels and ages (where this is the case) would also increase older adults’ confidence in trying out physical activity opportunities. While many older adults can navigate websites competently, they expressed a preference for simple information and were put off by too many moving images and the need to click through multiple links to find information.

### Conclusions

This study has provided insight into what older adults notice and look for in websites when exploring opportunities to increase their physical activity. It flagged how their pre-existing beliefs and implicit assumptions may influence whether they consider new options for activity, taking into account perceptions of their own capability, social norms, and some stereotypes around age and motivation for physical activity. These existing beliefs may interact with web content to exacerbate or potentially challenge stereotypes, to encourage older adults to feel more welcome to use mainstream facilities and services that support physical activity.

## Supplementary material

10.2196/68951 Multimedia Appendix 1 Supplementary materials containing the interview topic guide and a list of websites used in the study.
