# Supplementary material for: Exploring Older Adult’s Views of the Age-Inclusivity of Physical Activity Websites Using the Think Aloud Method: Qualitative Analysis
Source: JMIR Aging. 2025 Jun 19;8:e68951. doi: 10.2196/68951 (PMC12199841; doi:10.2196/68951)
Supplement: Multimedia Appendix 1 [file aging-v8-e68951-s001.docx]

**Supplementary data**

**Appendix 1: Interview schedule**

1. INTRODUCTION

- This is a study to develop understanding of older adults’ views on different sport industry websites and where they feel drawn to.
- I have a copy of each website here on my laptop and I will be showing you each of them and asking you to verbally describe what you’re thinking as you’re looking through the page. I will first ask you a few questions to understand your views on the sport industry and also ask you some questions as we go through the website and at the end, so I can get a good understanding about what you think about these websites.
- If you want to withdraw at any time and not take part anymore, please just let me know.
- If you are happy, we will record the interview on here, so we can listen again to what is being said. We will not keep anything on record that identifies you, or where you live or anyone else that you mention, so it will all be anonymous. Also, everything we talk about here will be confidential.
- Is there anything you would like to ask me at the moment?
- OK, so I will tell you a little more about what we will be doing today. We’re interested in your views on sport industry webpages. All you have to do is use the website as you would if I was not here and say your thoughts out loud. To help you think aloud you may find it useful to read aloud or tell me what you are clicking on and why. You may find at times I will say aloud what you have clicked on or what page you are looking at – this is just so when we listen to your views again we know what page you are talking about.
- This is not a test and you are not being judged. There are no right or wrong answers, so please say any thoughts which spring to mind, even if you think they might not be important. We just want you to say out loud any thoughts which are running through your mind.
- Please do feel free to say any negative thoughts you may have about the website as these will be really useful in helping us understand your views on it. I am not involved in any of the websites and am completely independent, so please do be honest. Your views about the website are really important so the more you can tell us the better.
- I won’t be able to answer your questions as we go through, but I can answer anything at the end. So if you ask me a question while you’re looking at the website, I will probably say that we can talk about it at the end.
- After you have finished looking at the website, I would like to have a chat with you about your overall views of the website.
- We can take a break at any time you like, please just let know and I can pause the recording. We can also stop the interview at any time if you want to.
- Ok so if you are happy, please can you complete this consent form?
- Do you have any questions? Are you happy if I start recording now?

1. WARM UP INTERVIEW

- I’m going to start by just asking you a few questions to help me understand your general views on exercise and the sport industry.
- Tell me about when you have exercised in the past – have you done it, and did you do it regularly?
  - Did you enjoy it?
  - Why did you participate in it?
  - Is there anything that made it hard for you to exercise?
- Do you exercise much now?
  - Why/why not?
  - What puts you off exercising?
- Do you feel welcome in sport settings such as gyms, equipment shops?
  - Why/why not?

1. THINK ALOUD AND RESEARCHER PROMPTS

- [only on first page] What are your first impressions of this page?
- What are you thinking now?
- What made you choose that option?
- What do you think about [this photo, this block of text..]?
- Can you tell me a bit more about why you think that?
- What is it you like about that?
- That’s really interesting./…..

1. POST THINK ALOUD QUESTIONS

- Overall, what do you think about the website?
- Can you tell me about anything you thought was particularly good about the website? (if needed)
- Can you tell me anything about the website that you were less keen on? (if needed)
- What do you think it would be like going to the gym/fitness shop/sport club in real life?
- Is there any aspect of the website that made you feel excluded or included?
  - Why did you feel excluded or included?
  - Was there any aspect of the website that made you feel included or excluded because of your age?
  - Is there anything that could be changed to make you feel more welcome?
- Would you go back to this website, and why?

1. THANK

COMPLETE SOCIODEMOGRAPHIC QUESTIONNIARE

GOODBYES

**Appendix 2: Websites used for the think aloud interviews**

All websites were presented to participants already open on a browser. The order in which they were presented was rotated between participants

All websites were last accessed 20/02/25.

1. Local authority providers of PA services/facilities/opportunities,

<https://www.better.org.uk/leisure-centre/banes/bath-sports-and-leisure-centre>

<https://www.sportwellsway.com/>

1. Fitness industry providers (e.g., gyms),

<https://www.puregym.com/>

<https://www.fitnessfirst.co.uk/>

1. Fitness industry product providers (e.g., equipment and clothing),

<https://www.johnmooresports.co.uk/>

<https://www.sportsdirect.com/>

1. Community organisations that encourage PA

<https://www.minervabathrc.org.uk/>

<https://www.bathramblingclub.co.uk/>

1. Campaigns

<https://www.ukactive.com/>

<https://wecanmove.net/>
